# The magnitude of intimate partner violence during pregnancy and associated factors in rural Ethiopia

**DOI:** 10.1093/inthealth/ihaf043

**Published:** 2025-04-17

**Authors:** Zeleke Dutamo Agde, Nega Assefa, Muluemebet Abera Wordofa

**Affiliations:** Department of Population Study and Family Health, Institute of Health, Jimma University, Jimma, P.O. Box +251 378, Ethiopia; Department of Reproductive Health, College of Medicine and Health Sciences, Wachemo University, Hossana, P.O. Box +251 667, Ethiopia; College of Health and Medical Sciences, Haramaya University, P.O. Box +251 138, Ethiopia; Department of Population Study and Family Health, Institute of Health, Jimma University, Jimma, P.O. Box +251 378, Ethiopia

**Keywords:** Hadiya Zone, intimate partner violence, physical violence, rural Ethiopia, sexual violence

## Abstract

**Background:**

Intimate partner violence (IPV) during pregnancy is a global public health issue associated with adverse maternal and newborn health outcomes. The aim of this study was to assess the magnitude of IPV during pregnancy and associated factors in rural Ethiopia.

**Methods:**

A cross-sectional survey was conducted among 432 pregnant women in the rural Hadiya Zone, Central Ethiopia, in July 2023, using structured interview questionnaires. Multivariable logistic regression was performed and the results were reported using adjusted ORs (AORs) with 95% CIs.

**Results:**

The overall prevalence of IPV during recent pregnancy was 38.37% (95% CI 33.82 to 43.18%). Among the specific forms of IPV, psychological, physical and sexual violence were 28.84% (95% CI 24.62 to 33.43%), 22.09% (95% CI 18.29 to 26.31%) and 20.70% (95% CI 17.02 to 24.84%), respectively. Key factors significantly associated with IPV during pregnancy included early marriage (before the age of 20 y), being uneducated, lower autonomy among women, husbands’ cigarette smoking and alcohol consumption, poor knowledge of IPV among husbands and husbands’ involvement in antenatal care (ANC). IPV during pregnancy was notably high in the study setting.

**Conclusions:**

Empowering women with low literacy, addressing male substance abuse, raising IPV awareness, promoting women's autonomy and encouraging male involvement in ANC visits are critical for reducing IPV.

## Introduction

Intimate partner violence (IPV) is one of the most prevalent forms of violence against women, and includes physical, sexual and emotional abuse by an intimate partner. One in three women may experience physical and/or sexual abuse in an intimate relationship.^1^ IPV is a human rights violation and a widespread public health concern.^2,3^

Being a victim of partner violence during pregnancy is a critical issue and can lead to adverse maternal and neonatal outcomes. Studies reveal that IPV is associated with adverse maternal outcomes, such as the risk of miscarriage, abortion, antepartum hemorrhage, hypertension, premature labor, gestational diabetes, placental problems, infections and mood disorders.^4,5^ Poor newborn outcomes, such as preterm birth, intrauterine fetal death, small gestational age and low birth weight, are associated with IPV.^6,7^ Research has linked IPV during pregnancy to mental disorders such as depression, anxiety and sleep disorders.^4,8^ Moreover, the use of maternal health services is inversely correlated with IPV. Evidence from 36 national household surveys in low- and middle-income countries by Leight and Wilson found that IPV decreases the utilization of maternal healthcare (antenatal care [ANC], skilled delivery and postnatal care).^9^ Furthermore, violence against women has adverse social and economic effects on the community and nation at large.^10,11^

Research has shown that younger age of women, higher age gap between couples, low educational status, being a housewife, partner’s alcohol use, unintended pregnancy and living in a rural setting are found to be the predictors of IPV during pregnancy.^12–17^

Various interventions have been implemented to prevent IPV against pregnant women and non-pregnant women, including screening and identification of IPV during ANC visits,^18^ start, awareness, support, action (or SASA),^19^ counseling,^20^ women empowerment,^21^ couples training,^22^ culturally appropriate interventions^23^ and father-focused interventions.^24^ A recent systematic and meta-analysis conducted to evaluate the effectiveness of community mobilization and group-based interventions in low- and middle-income countries has shown the promising effect of preventing IPV.^25^

Pregnancy is a very vulnerable period that has been linked to a higher risk of IPV.^24,26^ Studies conducted across different regions of Ethiopia have assessed the prevalence of IPV and its associated factors, reporting alarmingly high rates ranging from 26% to 65%, among the highest globally.^13–17^ Hadiya Zone is in a region with one of the high fertility rates^27^ and a significant proportion of men had a supportive attitude towards wife-beating under certain conditions, likely increasing the risk of IPV.^28^ However, limited evidence exists on the magnitude and associated factors of IPV specifically during pregnancy within rural settings. This study aimed to assess IPV during pregnancy and its associated factors within a specific rural setting. The findings aim to inform the development of an intervention strategy, given the limited evidence on interventions specifically designed to prevent IPV during pregnancy, particularly in rural Ethiopia.

## Materials and methods

### Study design, setting and period

A baseline cross-sectional survey was conducted among 432 pregnant women, which is part of the cluster randomized controlled trial that examines the effect of couple-based violence prevention education on IPV during pregnancy in rural districts of Hadiya Zone, Central Ethiopia. Hadiya Zone is one of the zones of the central Ethiopia regional state, Ethiopia. Its capital town, Hossana, is located 235 km southwest of the capital city, Addis Ababa. Hadiya Zone has four town administrations and 13 woredas (districts) with an area of 3542.66 square km and a population density of 444 people per square km. According to the 2021 Hadiya Zone Statistics Office report, the zone had a total population of 1 767 390, of whom 894 299 (50.6%) are females. The additional details of the study setting are reported in a separate publication.^29^ The study was conducted in Soro, Lemo, Analemo and Duna districts of Hadiya Zone, central Ethiopia. Data were collected during 1–30 July 2023.

### Study population, inclusion and exclusion criteria

Pregnant women in their first trimester (<13 wk of gestational age) and living with their husbands were included in the study. Couples where either the husband or wife planned to move out of the study area within the next 8 mo after baseline data collection were excluded from the study.

### Sample determination

The sample size was calculated using Stata version 16.0 (StataCorp LLC, College Station, TX, USA) by taking into account the following assumptions and parameters: an intracluster correlation coefficient of 0.05,^23^ a proportion (37.5%) of IPV during pregnancy from the study conducted in Ofla District, Ethiopia,^12^ an effect size of 0.2, a 95% CI, a power of 80% and an cluster size (m) of 27. The design effect of 2.3 is considered to account for the lack of independence between participants within clusters and to improve the power of the study. The design effect is defined as 1+ρ (m-1), where ρ is the intracluster correlation coefficient and m is the number of pregnant women per cluster. By taking into account a 20% loss to follow-up, a total of 432 pregnant women were included in the study.

### Sampling procedure

In this study, Kebele, the lowest administrative unit in Ethiopia, was considered as the cluster. A cluster sampling technique was used to select the participants. Soro, Lemo, Analemo and Duna districts were selected by simple random sampling from 13 districts found in Hadiya Zone. The selected districts comprised a total of 116 rural kebeles (clusters), of which 49 were identified as eligible for the study. Then, a simple random sampling technique was used to select 16 clusters out of 49 eligible clusters found in four districts. The census was conducted among 16 clusters to identify the households with eligible pregnant women. In each cluster, averages of 27 pregnant women were identified. Data were collected from all eligible women found in each cluster.

### Data collection

Structured interview questionnaires, which were adapted from a WHO multicountry study on women's health and domestic violence, were used to collect data.^30^ The questionnaires were pretested in a non-study kebele for clarity, logical flow and language appropriateness and subsequent amendments were made accordingly. The questionnaires were prepared in English then translated into the local language (Hadiyisa). Female supervisors and diploma-holder female nurses were utilized for supervision and data collection, respectively. The principal investigator trained the supervisors and data collectors. Data were collected using face-to-face interviews. The interview questionnaire had four parts: (i) sociodemographic and economic parts; (ii) women's autonomy; (iii) questions related to recent pregnancy and childbirth; and (iv) women's experience of IPV during the recent pregnancy.

### Outcome of interest and measurement

The outcome of the study concerns if there is IPV during a recent pregnancy. A pregnant woman was considered to have experienced IPV if she experienced at least one form of physical, psychological or sexual violence from her husband during her recent pregnancy. The measurement of IPV was based on the widely recognized WHO multicountry study on women's health and domestic violence against women, a comprehensive and validated framework used in similar research globally.^30^

Physical IPV was assessed by asking six physical IPV questions about whether her husband (i) slapped or threw something at her that could hurt her; (ii) pushed, shoved or pulled her hair; (iii) hit her with his fist or something else that could harm her; (iv) kicked, dragged or beat her up; (v) tried to choke her or burn her on purpose; and (vi) threatened to use or actually use a gun, knife or other weapon against her. If a pregnant woman responded ‘yes’ to one or more of these questions, it was recorded as yes=1 (experienced physical IPV); otherwise, no=0 (not experienced physical IPV).^30^

Sexual IPV was assessed by asking three sexual IPV questions: whether her husband (i) physically forced her to have sexual intercourse with him when she did not want to; (ii) forced her with threats or in any other way to perform sexual acts she did not want to; and (iii) physically forced her to perform any other sexual acts she did not want to during the recent pregnancy. If the pregnant woman responded ‘yes’ to one or more of these questions, it was recorded as yes=1 (experienced sexual IPV); otherwise, no=0 (not experienced any sexual IPV).^30^

Psychological IPV was assessed by asking four psychological violence questions: whether her husband (i) said or did something to humiliate her in front of another person; (ii) threatened to hurt or harm her or someone she cared about; (iii) insulted or made her feel bad about herself; and (iv) did things to scare or intimidate her on purpose. If the woman responded ‘yes’ to one of these questions, it was recorded as yes=1; otherwise, no=0 (not experienced emotional or psychological IPV during a recent pregnancy).^30^

The woman's decision-making autonomy was assessed by asking her who made decisions on household purchases, visiting relatives and friends, spending the wife's earnings, the number of children to have and obtaining her own healthcare. The woman scored one (1) for each variable that included her (made decisions alone or jointly with her husband); otherwise, she scored zero (0). Two binary measures were created to indicate higher autonomy (score of ≥3) vs lower autonomy (score ≤2).^31^ Five autonomy questions were evaluated for internal consistency, and the results showed a Cronbach alpha of 0.73, which was within an acceptable range.

Participants' knowledge status was assessed using nine IPV knowledge questions, with those scoring ≥5 of the correct answers considered to have good knowledge and those <5 considered to have poor knowledge.^32^ We assessed the internal consistency of nine knowledge questions and obtained a Cronbach alpha of 0.81, which was in an acceptable range.

Principal component analysis (PCA) was performed to generate a wealth index. Data were collected on various indicators of wealth, including housing type, ownership of household assets, livestock, farming land, as well as the production of crops, vegetables and fruits. These variables were used as input for the PCA. The PCA yielded three categories of household wealth index: low, medium and high economic status.

### Data processing and analysis

A complete dataset with individual codes and specific kebeles was exported from the server to SPSS version 25 for analysis. The study population was described using frequencies, percentages, means and SDs. The results are displayed in figures, diagrams and tables. A bivariate analysis was conducted to calculate the crude OR with a 95% CI to assess the association between dependent and independent variables. Variables for analysis were selected based on theoretical relevance and prior literature, and those with p<0.25 in the bivariable analysis were included in the multivariable logistic regression model. All tests were two-tailed, and statistical significance was declared at p<0.05. Hosmer and Lemeshow goodness-of-fit were used to assess the model's fitness, and its value was 0.86.

## Results

### Sociodemographic and economic characteristics of respondents

Out of 432 pregnant women included in the study, 430 responded properly, giving a response rate of 99.54%. Two hundred and seventy-seven (64.44%) of the women were within the age group of 25–34 y. The mean age of the women was 29.40 (SD 5.69) y. A majority (80.69%) of the respondents were married at age ≥20 y, with a mean and SD of 21.84 and 3.05, respectively. Two hundred and sixty-three (61.16%) of the respondents were Protestant Christian religion followers, while 21.63% were Orthodox Christians. Out of the total respondents, 64.18% were Hadiya, followed by Kembata (15.34%) and Silte (9.07%), by their ethnicity. Regarding women's literacy, 38.60% attended elementary school, 16.74% attended secondary education and 6.29% attended college and above education, whereas 38.37% had no formal education. A majority (77.44%) of the pregnant women were housewives, while 13.25% and 9.31% were merchants and employed, respectively (Table 1).

**Table 1.** Sociodemographic and economic characteristics of respondents in Hadiya Zone, Central Ethiopia, July 2023 (n=430)

Variable	Category	Frequency (n=430)	%
Age group of women, y	15–24	67	15.58
	25–34	277	64.44
	35–49	86	19.98
Age at marriage, y	<20	83	19.31
	≥20	347	80.69
Woman’s religion	Protestant Christian	263	61.16
	Orthodox Christian	93	21.63
	Other^a^	74	17.21
Woman’s ethnicity	Hadiya	276	64.18
	Kembata	66	15.34
	Silte	39	9.07
	Other^b^	49	11.41
Woman's education	No education	165	38.37
	Elementary school	166	38.60
	Junior or high school	72	16.74
	College/higher	27	6.29
Husband's education	No education	155	36.05
	Elementary school	137	31.86
	Junior or high school	80	18.60
	College/higher	58	13.49
Woman's occupation	Housewife	333	77.44
	Merchant	57	13.25
	Employed	40	9.31
Woman's autonomy	Higher	200	46.51
	Lower	230	53.49
Household wealth index	Low	143	33.25
	Medium	144	33.50
	High	143	33.25

a Amhara, Gurage, Oromo, Wolayita.

b Muslim, Catholic, Adventist, Apostolic.

### Pregnancy and reproductive history of respondents and male partner lifestyle

More than one-third (33.25%) of the pregnancies were unintended; of these, 20.93% were mistimed, and 12.32% were unwanted. Approximately one-half (49.07%) of pregnant women had ≥5 live births prior to their current pregnancy, while 11.16% had one live birth. The mean and SD of the live births were 4.63 ± 2.26, with a range of 1 and 11 live births. Seven in 10 pregnant women (70.23%) had received ANC from a skilled healthcare provider at least once for their recent pregnancy. Out of 302 pregnant women who attended ANC, 40.69% were accompanied at least once during ANC visits by their husbands. Respondents’ husbands had substance abuse behavior; 27.21% drank alcohol and 18.84% smoked cigarettes.

### Prevalence of IPV and its forms

**Figure 1.**
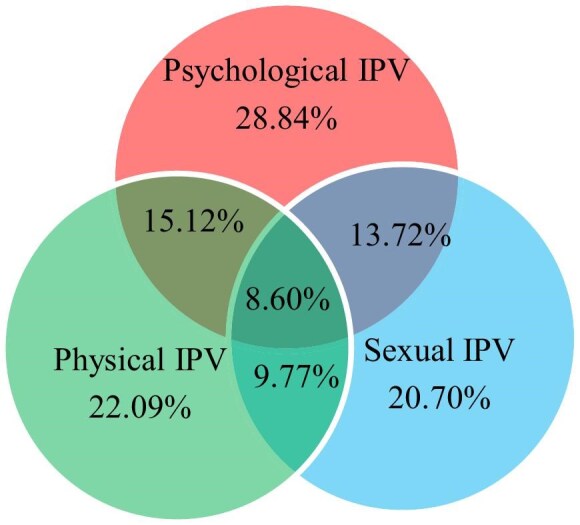
Forms of intimate partner violence (IPV) during recent pregnancy in rural districts of Hadiya Zone, Central Ethiopia, July 2023 (n=430).

About four in 10 women (38.37%; 95% CI 33.82 to 43.18%) experienced at least one form of IPV during a recent pregnancy. Psychological IPV was the most prevalent (28.84%; 95% CI 24.62 to 33.43%) form of IPV, followed by 22.09% (95% CI 18.29 to 26.31%) and 20.70% (95% CI 17.02 to 24.84%) physical and sexual IPV, respectively. Respondents experienced some forms of violence simultaneously. Among the participants, 15.12% experienced both physical and psychological IPV, while 13.72% experienced both psychological and sexual IPV. Out of all the pregnant women who participated in the study, 8.60% were the victims of all three forms of IPV (physical, psychological and sexual) (Figure 1).

The most frequently experienced psychological or emotional violence by pregnant women was intimidation to scare or intimidation on purpose (19.30%), whereas the least frequently experienced was threatening to hurt or harm her or someone she cares about (9.77%). Among the physical violence experienced by the respondents, slapping or throwing that hurt was the most frequent (15.12%), while choking or burning on purpose was the least frequent (4.19%). The most frequently experienced sexual IPV by the respondents was being forced with threats or in other ways to engage in sexual acts without the respondent’s willingness (Table 2).

**Table 2.** The responses regarding intimate partner violence during recent pregnancy in rural districts of Hadiya Zone, Central Ethiopia, July 2023 (n=430)

Items of violence	Frequency (n)	%
Psychological intimate partner violence	**124**	**28.84**
Did things to intimidate to scare or intimidated on purpose	83	19.30
Insulted or made you feel bad about yourself	59	13.72
Threatened to hurt or harmed you or someone you care	42	9.77
Belittled or humiliated in front of others	50	11.63
Physical intimate partner violence	**95**	**22.09**
Slapped or threw something that could hurt you	65	15.12
Pushed or shoved or pulled your hair	42	9.77
Hit with fist or something that could hurt you	36	8.37
Kicked or dragged/beat up	29	6.74
Threatened to use or used a gun, knife or any other weapon against you	23	5.35
Choked or burned on purpose	18	4.19
Sexual intimate partner violence	**89**	**20.70**
Forced you with threats or in any other way to have sexual acts you did not want	55	12.79
Physically forced to have any other sexual acts you did not want	49	11.39
Physically forced to have sexual intercourse you did not want to	43	10.00

### Factors associated with IPV during pregnancy

After adjustment for covariates, woman's age at marriage, woman's education, woman's autonomy, husband’s knowledge of IPV, husband’s smoking, husband’s alcohol drinking and husband’s involvement in ANC, were significantly associated with IPV during recent pregnancy. The odds of experiencing IPV in recent pregnancy were 2.21 (AOR=2.21; 95% CI 1.17 to 4.15) times higher among women married before the age of 20 y compared with women married at the age of ≥20 y. Educational status was inversely related to experience of IPV during pregnancy. Pregnant women with elementary education were 69% (AOR=0.31; 95% CI 0.18 to 0.55) less likely to experience IPV during pregnancy compared with women with no education. Similarly, women with high school education were 81% (AOR=0.19; 95% CI 0.08 to 0.43) and women with college/higher education were 84% (AOR=0.16; 95% CI 0.04 to 0.60) less likely to experience IPV during pregnancy compared with women with no education. Women with lower household decision-making autonomy were 3.82 (AOR=3.82; 95% CI 2.23 to 6.57) times more likely to experience IPV compared with their counterparts.

The husband's alcohol consumption increased the odds of experiencing IPV 3.69 (AOR=3.69; 95% CI 2.03 to 6.71) times among pregnant women. Pregnant women whose husbands smoked cigarettes were 2.31 (AOR=2.31; 95% CI 1.14 to 4.65) times more likely to experience IPV compared with those whose husbands did not smoke. A husband’s knowledge of IPV was significantly associated with a pregnant woman's experience of IPV. Pregnant women whose husbands had a poor knowledge of IPV were 2.65 (AOR=2.65; 95% CI 1.58 to 4.44) times more likely to experience IPV compared with those husbands had a good knowledge of IPV. Pregnant women with husbands who accompanied them during an ANC visit were 75% (AOR=0.25; 95% CI 0.15 to 0.44) less likely to experience IPV compared with those who husbands did not accompany them (Table 3).

**Table 3.** Factors associated with IPV during recent pregnancy in rural districts of Hadiya Zone, central Ethiopia, July 2023 (N=430)

Explanatory variable	Category	N	COR (95% CI)	AOR (95% CI)
Women age group, y	15–24	69	1.48 (0.78 to 2.82)	1.48 (0.51 to 4.27)
	25–34	275	0.83 (0.51 to 1.37)	1.35 (0.68 to 2.70)
	35–49	86	1.00	1.00
Woman's age at marriage, y	<20	83	2.11 (1.30 to 3.42)^*^	2.21 (1.17 to 4.15)^*^
	≥20	341	1.00	1.00
Woman's education	No education	165	1.00	1.00
	Elementary school	166	0.26 (0.16 to 0.41)^***^	0.31 (0.18 to 0.55)^***^
	High school	72	0.11 (0.05 to 0.23)^***^	0.19 (0.08 to 0.43)^***^
	College/higher	27	0.08 (0.02 to 0.27)^***^	0.16 (0.04 to 0.60)^***^
Husband's education	No education	155	1.00	1.00
	Elementary school	137	0.75 (0.47 to 1.18)	1.01 (0.54 to 1.87)
	High school	80	0.33 (0.18 to 0.60)^***^	0.67 (0.31 to 1.44)
	College/higher	58	0.16 (0.07 to 0.35)^***^	0.40 (0.15 to 1.09)
Social support	Poor	288	1.00	1.00
	Moderate	99	0.70 (0.43 to 1.11)	0.89 (0.46 to 1.70)
	Strong	43	0.47 (0.23 to 0.98)^*^	0.61 (0.25 to 1.51)
Husband smokes cigarettes	Yes	81	3.74 (2.25 to 6.21)^***^	2.31 (1.14 to 4.65)^*^
	No	349	1.00	1.00
Husband drinks alcohol	Yes	117	5.19 (3.29 to 8.20)^***^	3.69 (2.03 to 6.71)^***^
	No	313	1.00	1.00
Woman's autonomy	Higher	200	1.00	1.00
	Lower	230	3.76 (2.47 to 5.72)^***^	3.82 (2.23 to 6.57)^***^
Pregnancy intention	Intended	287	1.00	1.00
	Unintended	143	1.70 (1.13 to 2.56)^*^	1.1 (0.6 to 2.0)
Parity	1	48	0.68 (0.35 to 1.31)	0.70 (0.28 to 1.72)
	2–4	171	0.57 (0.38 to 0.88)^*^	0.75 (0.43 to 1.31)
	≥5	211	1.00	1.00
Husband's knowledge of IPV	Good	248	1.00	1.00
	Poor	182	3.62 (2.41 to 5.45)^***^	2.65 (1.58 to 4.44)^***^
Husband's involvement in ANC	Yes	175	0.22 (0.14 to 0.34)^***^	0.25 (0.15 to 0.44)^***^
	No	255	1.00	1.00
Household wealth index	Low	143	1.00 (0.62 to 1.62)	1.21 (0.63 to 2.32)
	Medium	144	1.28 (0.80 to 2.07)	1.54 (0.81 to 2.92)
	High	143	1.00	1.00

ANC: antenatal care; AOR: adjusted OR; COR: crude OR; IPV: intimate partner violence.

* p<0.05.

*** p<0.001.

## Discussion

This study revealed that the overall prevalence of IPV during recent pregnancy was 38.37%. This finding was consistent with previous IPV studies in other parts of Ethiopia, Ofla District^12^ and Harar District.^33^ The magnitude of IPV during pregnancy in the study setting was high. A recent systematic review and meta-analysis conducted by Leight et al. showed that community mobilization and group-based interventions in low- and middle-income countries are effective in preventing IPV.^25^ Moreover, a recent systematic review has shown that father-centered interventions are effective in preventing or reducing IPV during pregnancy and early parenthood.^24^ The high burden of IPV during pregnancy in the community underscores the urgent need for targeted interventions, such as couple-based violence prevention education, to address the harmful consequences of IPV, challenge norms that normalize violence and improve conflict resolution skills.^22^

The odds of experiencing IPV in recent pregnancy were about two times higher among women married before the age of 20 y compared with women married at the age of ≥20 y. A similar association was found in other studies.^34,35^ This could be because women who married at an early age may be prone to power imbalance, decreased autonomy in household decision-making, a lower level of education attained and the stress associated with becoming mothers at a young age. This finding implies that interventions aimed at discouraging early marriage can help reduce women's vulnerability to IPV during pregnancy by promoting their autonomy, education and decision-making power. In this study, education status was inversely associated with IPV. A similar association was found in other studies.^13,36^ Addressing uneducated women in this community through couples-based education can create meaningful prevention or reduction of IPV during pregnancy.

The alcohol drinking habits of respondents’ partners were found to be a predictor of IPV, indicating that pregnant women with husbands who drank alcohol were about four times more likely to be victims of IPV compared with those with non-drinking partners. This finding corroborates with other studies conducted in Ethiopia.^12–14,33,37,38^ This may be due to the fact that alcohol can impair judgment and self-control, exacerbate existing stress, cause aggression and increase the likelihood of violent behavior. This implies that reducing IPV during pregnancy might be addressed by educating couples about the negative effects of alcohol usage in relationships. This survey revealed that pregnant women with husbands who smoked cigarettes had a twofold higher chance of experiencing IPV. This result is consistent with prior research.^14,39^ The possible justification for this could be that substance dependence may lead to mood changes and irritability, potentially resulting in unstable relationships and perpetration. Women who had lower autonomy in household decision-making were more likely experience IPV during pregnancy. Similar findings were observed in other studies.^15,40^

Pregnant women whose husbands had a poor knowledge of IPV were about three times more likely to experience IPV compared with those whose husbands had a good understanding of IPV. This shows that enhancing knowledge and awareness among husbands about IPV and its consequences might potentially reduce the risk of violence against pregnant women. A husband’s involvement during the ANC visit was negatively associated with IPV. Pregnant women without husbands accompanying them during an ANC visit were four times more likely to experience IPV compared with those who were accompanied. This suggests that efforts to engage husbands in ANC and change the norms that condone violence might be crucial in preventing IPV.

This baseline assessment had some limitations. A major limitation of this study is its cross-sectional design, which limits the ability to infer causal relationships between IPV and its associated factors. The topic is very sensitive; there might be social desirability bias not to disclose such private experiences, resulting in an under-reporting of IPV. We tried to maximize disclosures using female supervisors and data collectors according to WHO ethical guidelines.^41^ Recall bias also may be a potential limitation of this study because the women were asked about their experiences of IPV during a recent pregnancy within the last 2 y.

## Conclusions

This study found that 38.37% of women experienced IPV during their most recent pregnancy, a prevalence that is relatively high. Several factors were significantly associated with IPV during pregnancy, including early marriage (before the age of 20 y), being uneducated, limited autonomy in household decision-making, the husband's alcohol consumption and cigarette smoking, poor knowledge of IPV among husbands and male involvement in ANC visits. These findings underscore that targeted interventions should prioritize women with low literacy levels, address substance abuse among men, increase awareness and knowledge about IPV and its consequences among husbands, enhance women's autonomy and challenge harmful norms related to IPV.

## Data Availability

The data underlying this article will be shared on reasonable request to the corresponding author.
